# Species distribution of nontuberculous mycobacteria isolated from respiratory specimens at a tertiary care hospital in South Korea, 2017–2022

**DOI:** 10.1128/spectrum.00554-25

**Published:** 2025-09-17

**Authors:** Tae Yeul Kim, Jeong Su Park, Minhee Kang, Byung Woo Jhun, Hee Jae Huh, Nam Yong Lee, Sue Shin

**Affiliations:** 1 Department of Laboratory Medicine and Genetics, Samsung Medical Center, Sungkyunkwan University School of Medicine, Seoul, South Korea; 2 Department of Laboratory Medicine, Seoul National University Bundang Hospital 65462, Seongnam, South Korea; 3 Department of Laboratory Medicine, Seoul National University College of Medicine, Seoul, South Korea; 4 Biomedical Engineering Research Center, Smart Healthcare Research Institute, Samsung Medical Center, Seoul, South Korea; 5 Division of Pulmonary and Critical Care Medicine, Department of Medicine, Samsung Medical Center, Sungkyunkwan University School of Medicine, Seoul, South Korea; 6 Department of Medical Device Management and Research, Samsung Advanced Institute for Health Sciences & Technology, Sungkyunkwan University, Seoul, South Korea; 7 Department of Laboratory Medicine, Seoul Metropolitan Government-Seoul National University Boramae Medical Center, Seoul, South Korea; Assistance Publique - Hopitaux de Paris Universite Paris Saclay, Clamart, France

**Keywords:** nontuberculous mycobacteria, species distribution, respiratory specimens, Korea

## Abstract

The clinical relevance and drug resistance patterns of nontuberculous mycobacteria (NTM) vary by species. This study investigated the species distribution of NTM isolated from respiratory specimens at a tertiary care hospital in South Korea from 2017 to 2022. A retrospective analysis was conducted on laboratory data from respiratory specimens submitted for acid-fast bacilli culture. NTM isolates were identified using a line probe assay, and those unidentifiable at the species or complex level underwent multigene sequencing of the 16S rRNA, *rpoB*, and *hsp65* genes. Among all mycobacterial isolates, the proportion of NTM showed an increasing trend, rising from 87.4% in 2017 to 93.3% in 2022. The eight most common species were *M. avium* complex (61.9%), *M. abscessus* (14.2%), *M. fortuitum* complex (8.4%), *M. gordonae* (5.3%), *M. simiae* complex (3.4%), *M. kansasii* complex (1.9%), *M. terrae* complex (1.5%), and *M. chelonae* (1.2%), accounting for 97.7% of all NTM isolates. Among the remaining isolates (2.3%, *n* = 169), 161 were classified into 24 species and groups, the majority with proportions below 0.1%. Two of the eight isolates that could not be identified at the species or group level despite multigene sequencing underwent whole-genome sequencing, which suggested they likely represent novel *Mycobacterium* species. This study provides valuable insights into the distribution of NTM species, particularly rarely encountered species, isolated from respiratory specimens in South Korea. These findings may aid in optimizing diagnostic strategies and selecting appropriate treatment options.

**IMPORTANCE:**

Given the significant variations in clinical relevance and drug resistance patterns among nontuberculous mycobacteria (NTM) species, understanding their geographic distribution is essential for selecting appropriate treatment options and improving patient outcomes. This study investigated the distribution of NTM species isolated from respiratory specimens at a tertiary care hospital in South Korea from 2017 to 2022. Our findings revealed an increasing proportion of NTM, with *M. avium* complex and *M. abscessus* remaining predominant. Additionally, we identified 24 rarely encountered species and groups, along with two strains that likely represent novel *Mycobacterium* species. Our study advances the understanding of the evolving NTM epidemiology in South Korea, contributing to the optimization of diagnostic strategies and improvement of patient management.

## INTRODUCTION

Nontuberculous mycobacteria (NTM) pulmonary disease is an increasingly significant public health concern worldwide due to its rising prevalence (1–3) and poor long-term outcomes (4–7). The distribution of NTM species isolated from respiratory specimens varies geographically (8–16). Given that the clinical relevance and drug resistance patterns of NTM differ significantly by species (9, 17–24), understanding the geographic distribution of NTM species is crucial for selecting appropriate treatment options and improving patient outcomes.

Line probe assays (LPAs), such as INNO-LiPA MYCOBACTERIA (Fujirebio Europe, Ghent, Belgium) and GenoType Mycobacterium CM/AS (Hain Lifescience, Nehren, Germany), are widely used in clinical microbiology laboratories. LPAs are simple to perform and provide accurate identification of commonly encountered NTM species, such as *M. avium*, *M. intracellulare*, and *M. abscessus* (25). However, LPAs have inherent limitations, including a restricted number of probes and the potential for cross-hybridization with unrelated species, which may lead to the failure to identify rarely encountered NTM species at the species or complex level or result in their misidentification (26–28). Thus, knowledge of the geographic distribution of NTM species, especially the rarely encountered ones, is essential for selecting the most suitable LPAs for each region.

In South Korea, several studies have explored the distribution of NTM species isolated from respiratory specimens. However, these studies often included a limited number of isolates or relied on species identification methods with low discriminatory power, such as LPAs, high-performance liquid chromatography, and PCR-restriction fragment length polymorphism, resulting in insufficient data on rarely encountered NTM species (10, 11, 15, 29–33). Samsung Medical Center, a 2,000-bed tertiary care hospital in Seoul, South Korea, serves a large number of NTM patients nationwide. In this study, we analyzed the species distribution of 7,397 NTM isolates from respiratory specimens submitted to the Clinical Microbiology Laboratory of Samsung Medical Center between 2017 and 2022. During this period, multigene sequencing of the 16S rRNA, *rpoB*, and *hsp65* genes was performed on isolates unidentifiable at the species or complex level by an LPA (AdvanSure Mycobacteria GenoBlot Assay; Invitros, Seoul, South Korea), enabling the analysis of the proportions of rarely encountered NTM species.

The objective of this study was to analyze recent trends in NTM isolation from respiratory specimens and to assess species distribution, with a particular focus on rarely encountered species and regional variation, at a tertiary care hospital in South Korea.

## MATERIALS AND METHODS

### Data collection

Laboratory data from respiratory specimens submitted for acid-fast bacilli (AFB) culture to the Clinical Microbiology Laboratory of Samsung Medical Center between January 2017 and December 2022 were extracted from the laboratory information system. These data included results from AFB culture and species identification. For cases in which NTM were isolated from AFB cultures, patients’ addresses were retrieved and categorized into five regions: (i) Seoul Capital Area (SCA), comprising Seoul City, Incheon City, and Gyeonggi Province; (ii) Gangwon Region, consisting of Gangwon Province; (iii) Chungcheong Region, encompassing North Chungcheong Province, South Chungcheong Province, Daejeon City, and Sejong City; (iv) Gyeongsang Region, covering North Gyeongsang Province, South Gyeongsang Province, Busan City, Daegu City, and Ulsan City; and (v) Jeolla Region, incorporating North Jeolla Province, South Jeolla Province, Jeju Province, and Gwangju City.

### AFB culture

Respiratory specimens were processed using the *N*-acetyl-*L*-cysteine–NaOH method and inoculated into mycobacterial growth indicator tubes (BD, Sparks, MD, USA) and 3% Ogawa agar (Shinyang, Seoul, South Korea), followed by incubation for 6 weeks. All positive cultures underwent Ziehl-Neelsen staining to confirm the presence of AFB. For cultures confirmed as AFB-positive, DNA was extracted using the boiling method, and real-time PCR was performed using the GREENCARE MTB/NTM detection kit (GC Medical Science, Yongin, South Korea), which distinguishes *M. tuberculosis* complex (MTBC) from NTM, following the manufacturer’s instructions. Briefly, 5 µL of extracted DNA was added to 15 µL of master mix containing primers and probes, resulting in a total reaction volume of 20 µL. Amplification was performed on an Applied Biosystems 7500 Real-Time PCR System (Thermo Fisher Scientific, Waltham, MA, USA) using the following cycling conditions: 95°C for 15 min, followed by 40 cycles of 95°C for 15 s and 60°C for 30 s. The assay targets the *IS6110* region for MTBC detection (FAM channel) and the 16S rRNA gene as a pan-*Mycobacterium* target for NTM detection (VIC channel). A culture was classified as MTBC-positive when the MTBC target was positive (cycle threshold [Ct] value ≤ 40) and its Ct value was lower than that of the pan-*Mycobacterium* target. If the Ct value of the MTBC target was higher than that of the pan-*Mycobacterium* target, the culture was classified as positive for both MTBC and NTM. A culture was considered NTM-positive when the pan-*Mycobacterium* target was positive (Ct value ≤ 35) and the MTBC target was negative.

### Species identification

NTM isolates confirmed by real-time PCR were subjected to species identification using the AdvanSure Mycobacteria GenoBlot Assay at the attending physician’s request. This assay targets the internal transcribed spacer region and allows the simultaneous identification of 22 mycobacterial species and complexes, including MTBC, *M. avium*, *M. intracellulare*, *M. abscessus*, *M. chelonae*, *M. fortuitum* complex, *M. peregrinum*, *M. kansasii*, *M. gordonae*, *M. lentiflavum*/*M. genavense*, *M. terrae* complex, *M. simiae*, *M. scrofulaceum*, *M. celatum*, *M. malmoense*, *M. gastri*, *M. flavescens*, *M. vaccae*, *M. xenopi*, *M. smegmatis*, *M. szulgai*, and *M. marinum*/*M. ulcerans*. Previous studies have demonstrated that this assay performs well in identifying common NTM species and shows comparable accuracy to the widely used GenoType Mycobacterium CM/AS assay (34, 35). The assay was performed according to the manufacturer’s instructions. Briefly, DNA was extracted using the boiling method. Each PCR reaction contained 7.5 µL of extracted DNA, 12.5 µL of 2× reaction mixture, and 5 µL of primer mixture, resulting in a total volume of 25 µL. Amplification was carried out on an Applied Biosystems 7500 Real-Time PCR System using the following cycling conditions: 50°C for 2 min and 95°C for 10 min, followed by 15 cycles of 94°C for 30 s, 65°C for 1 min, and 72°C for 30 s; then 38 cycles of 94°C for 30 s, 55°C for 1 min, and 72°C for 30 s; and a final elongation step at 72°C for 10 min. PCR products were then subjected to hybridization, washing, and staining. Band intensities and patterns on the strip were detected and interpreted using the AdvanSure GenoLine Scan (Invitros). NTM isolates identified as *M. abscessus* were further analyzed using the ERM-plus real-time PCR kit (Invitros), which distinguishes *M. abscessus* subsp. *abscessus* from *M. abscessus* subsp. *massiliense* by detecting the erythromycin ribosome methyltransferase (*erm*) gene.

NTM isolates that could not be identified at the species or complex level by the AdvanSure Mycobacteria GenoBlot Assay were further analyzed through multigene sequencing of the 16S rRNA, *rpoB*, and *hsp65* genes. Genomic DNA was extracted using the MagNA Pure 96 System (Roche Diagnostics, Basel, Switzerland). PCR amplification was performed using the following primer sets: for the 16S rRNA gene, forward 5′- GAGAATTCGTGCTTAACACATGCAAGTCG-3′, reverse 5′- ATGGATCCGTGAGATTTCACGAACAACGC-3′ (36); for the *rpoB* gene, forward 5′-GGCAAGGTCACCCCGAAGGG-3′, reverse 5′-AGCGGCTGCTGGGTGATCATC-3′ (37); and for the *hsp65* gene, forward 5′- ACCAACGATGGTGTGTCCAT-3′, reverse 5′-CTTGTCGAACCGCATACCCT-3′ (38). PCR cycling conditions were as follows: 90°C for 10 min; 40 cycles of 94°C for 25 s, 60°C for 30 s, and 72°C for 45 s; followed by a final extension step at 72°C for 10 min. PCR products were sequenced using the same primers used for amplification. The obtained sequences were compared with those in the GenBank database using the Basic Local Alignment Search Tool (BLAST; https://blast.ncbi.nlm.nih.gov/Blast.cgi) and interpreted according to the criteria outlined in the Clinical and Laboratory Standards Institute guideline MM18, second ed (39). Species identification methods remained consistent throughout the study period.

### Whole-genome sequencing analysis

NTM isolates unresolved at the species or complex level by multigene sequencing were further analyzed using whole-genome sequencing (WGS) to determine their taxonomic status. Genomic DNA was extracted as described by Bouso et al. (40), and WGS was performed on the HiSeq X Ten (Illumina, San Diego, CA, USA) and GridION (Oxford Nanopore Technology, Oxford, UK) platforms. Nanopore reads were assembled using Canu v2.1.1 (41) and polished with Pilon v1.24 using Illumina reads (42), or alternatively, Nanopore and Illumina reads were hybrid-assembled using Unicycler v0.4.9 (43). The assembled genomes were annotated using Prokka v1.14.6 (44) and visualized using Proksee (45). The taxonomic status of these isolates was determined by calculating average nucleotide identity (ANI) and digital DNA-DNA hybridization (dDDH) values. ANI values were calculated using the OrthoANIu algorithm on the EzBioCloud server (https://www.ezbiocloud.net/) (46), and dDDH values were calculated using formula *d4* on the Type (Strain) Genome Server (https://tygs.dsmz.de/) (47).

### Data analysis

In calculating the distribution of NTM species, multiple species isolated from a single specimen were counted as distinct isolates. For patients with repeated isolation of the same species, only the first isolate was included, excluding duplicate isolates. The Chi-square test for trend was used to assess annual changes in the proportion of NTM among all mycobacterial isolates from respiratory specimens and in the proportions of major NTM species. A *P* value of <0.05 was considered statistically significant. Statistical analyses were conducted using Excel (Microsoft, Redmond, WA, USA) and Epi Info 7 (Centers for Disease Control and Prevention, Atlanta, GA, USA).

## RESULTS

### Annual trend in the proportion of NTM isolates

**Fig 1:**
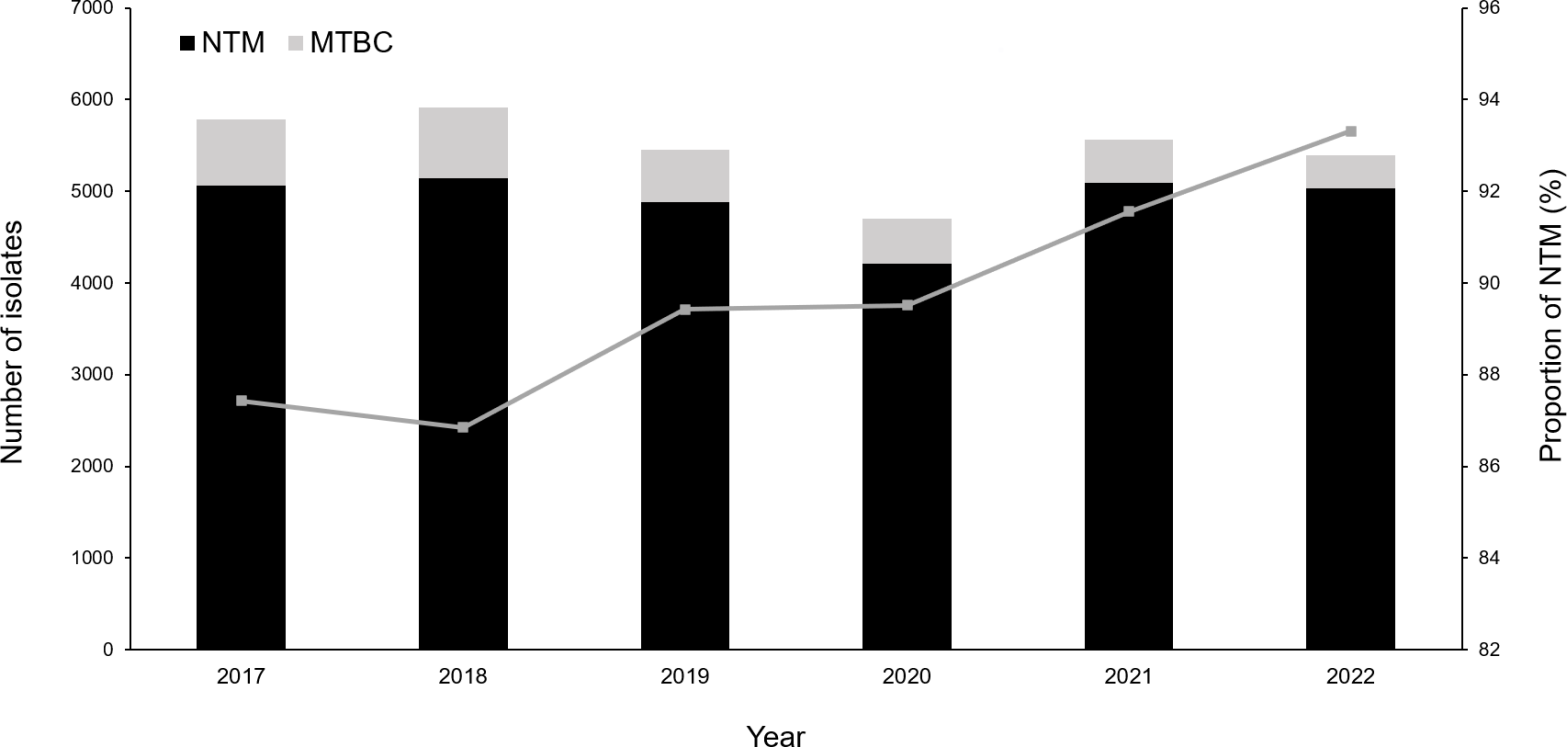
Annual trend in mycobacterial isolates from respiratory specimens. The left y-axis shows the number of isolates, with bars indicating the annual counts of MTBC (gray) and NTM (black). The right y-axis represents the proportion of NTM among all mycobacterial isolates, depicted by the line.

During the study period, a total of 147,543 respiratory specimens from 38,797 patients were submitted for AFB culture. The most common specimen type was sputum (89.0%), followed by bronchial wash or bronchoalveolar lavage fluid (10.0%), endotracheal aspirate (0.9%), lung aspirate (0.05%), and other specimen types (0.07%). Of these specimens, 67.0% were collected from outpatients and 33.0% from inpatients. According to AFB culture results, 3,370 (2.3%) were positive for MTBC, 29,365 (19.9%) for NTM, and 35 (0.02%) for both MTBC and NTM. Consequently, NTM accounted for 89.6% (29,400/32,805) of all mycobacterial isolates. Although the number of NTM isolates did not increase, the proportion of NTM among all mycobacterial isolates exhibited an overall increasing trend, rising from 87.4% (5,057/5,784) in 2017 to 93.3% (5,032/5,393) in 2022 (Fig. 1). This trend was statistically significant (*P* < 0.001).

### Distribution of NTM species

**Fig 2:**
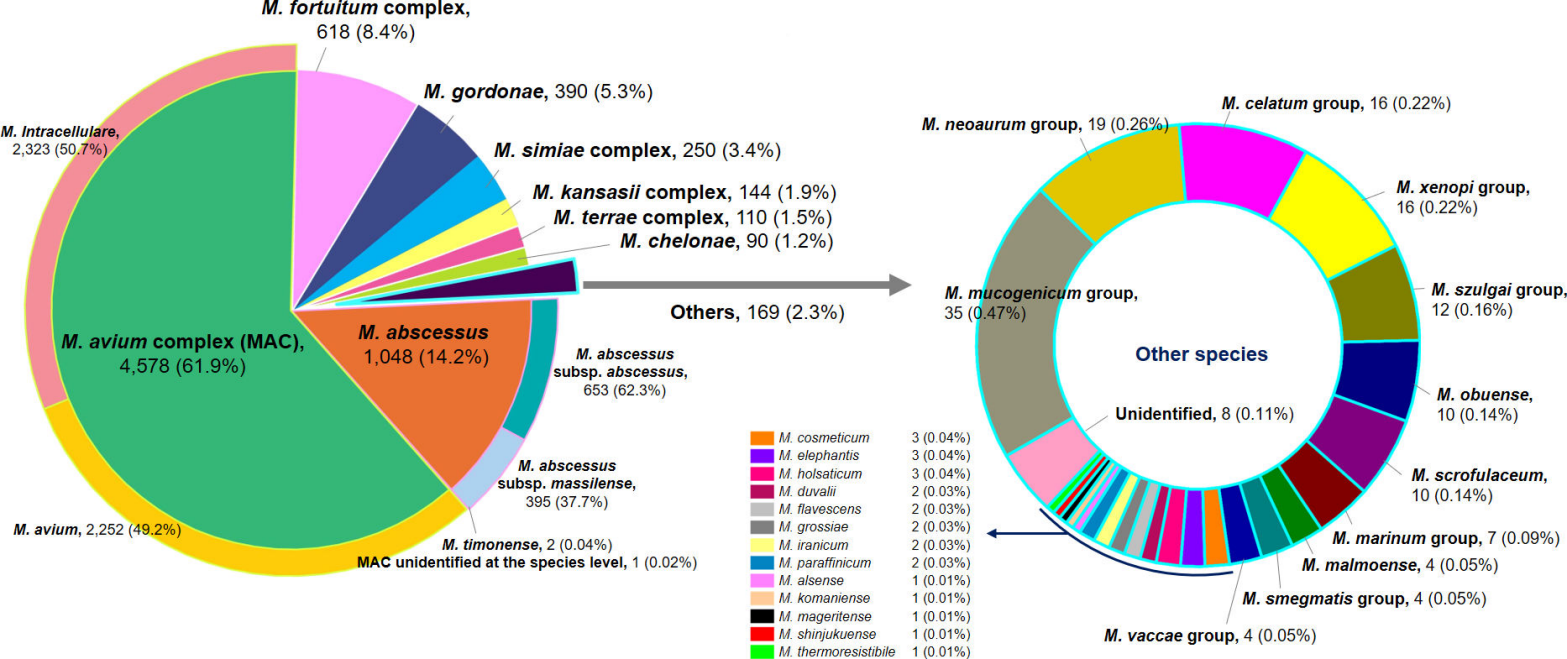
Species distribution of NTM isolates from respiratory specimens during 2017–2022.

**Fig 3:**
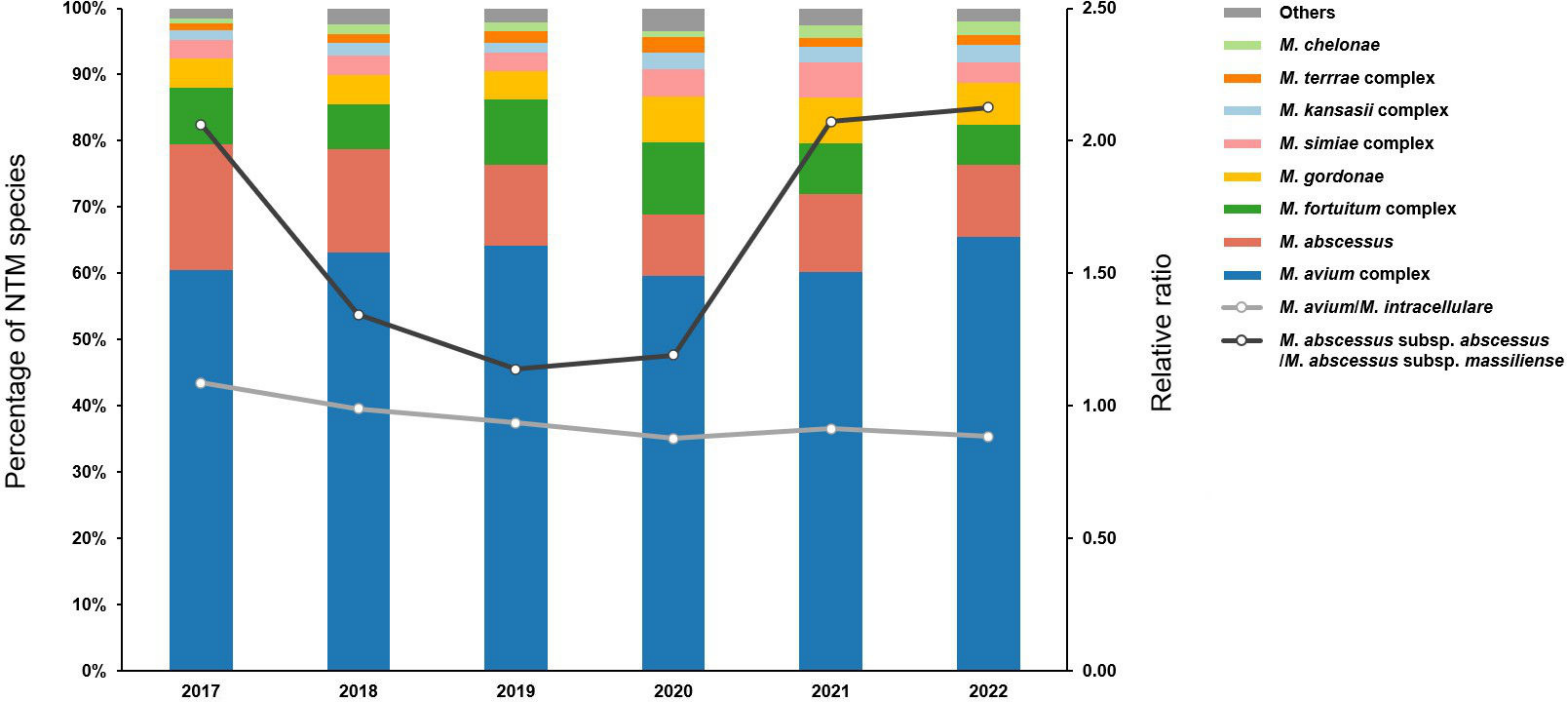
Annual trends in the proportions of the eight major NTM species. The left y-axis shows the proportion of each NTM species, with bars representing their annual proportions. The right y-axis shows relative ratios, with lines indicating the ratio of *M. avium* to *M. intracellulare* (light gray) and *M. abscessus* subsp. *abscessus* to *M. abscessus* subsp. *massiliense* (dark gray).

Among the 29,400 NTM isolates grown on AFB culture, 22,425 (76.3%) from 5,369 patients underwent species identification at the request of the attending physician. After counting multiple species from a single specimen as distinct isolates and excluding duplicate isolates from the same patients, 7,397 NTM isolates were included in the species distribution analysis. The eight most commonly isolated NTM were *M. avium* complex (61.9%, *n* = 4,578), *M. abscessus* (14.2%, *n* = 1,048), *M. fortuitum* complex (8.4%, *n* = 618), *M. gordonae* (5.3%, *n* = 390), *M. simiae* complex (3.4%, *n* = 250), *M. kansasii* complex (1.9%, *n* = 144), *M. terrae* complex (1.5%, *n* = 110), and *M. chelonae* (1.2%, *n* = 90), accounting for 97.7% of all NTM isolates (Fig. 2). Trends in the proportions of these major species during the study period are shown in Fig. 3. No significant trends were observed in the proportions of *M. avium* complex, *M. fortuitum* complex, and *M. terrae* complex (*P* = 0.428, *P* = 0.561, and *P* = 0.175, respectively). In contrast, the proportion of *M. abscessus* showed a significant decreasing trend (*P* < 0.001), while those of *M. gordonae*, *M. simiae* complex, *M. kansasii* complex, and *M. chelonae* exhibited significant increasing trends (*P* < 0.001, *P* = 0.008, *P* = 0.010, and *P* = 0.002, respectively). Within the *M. avium* complex, *M. intracellulare* was the most frequently isolated species (50.7%, *n* = 2,323), followed by *M. avium* (49.2%, *n* = 2,252), *M. timonense* (0.04%, *n* = 2), and one isolate (0.02%) that could not be identified at the species level (Fig. 2). The relative ratio of *M. avium* to *M. intracellulare* was 0.97 and exhibited a significant decreasing trend over the study period (*P* = 0.013), declining from 1.09 in 2017 to 0.88 in 2022 (Fig. 3). Within *M. abscessus*, *M. abscessus* subsp. *abscessus* (62.3%, *n* = 653) was isolated 1.65 times more frequently than *M. abscessus* subsp. *massiliense* (37.7%, *n* = 395) (Fig. 2). The relative ratio of *M. abscessus* subsp. *abscessus* to *M. abscessus* subsp. *massiliense* showed no significant trend during the study period (*P* = 0.681) (Fig. 3).

**Fig 4:**
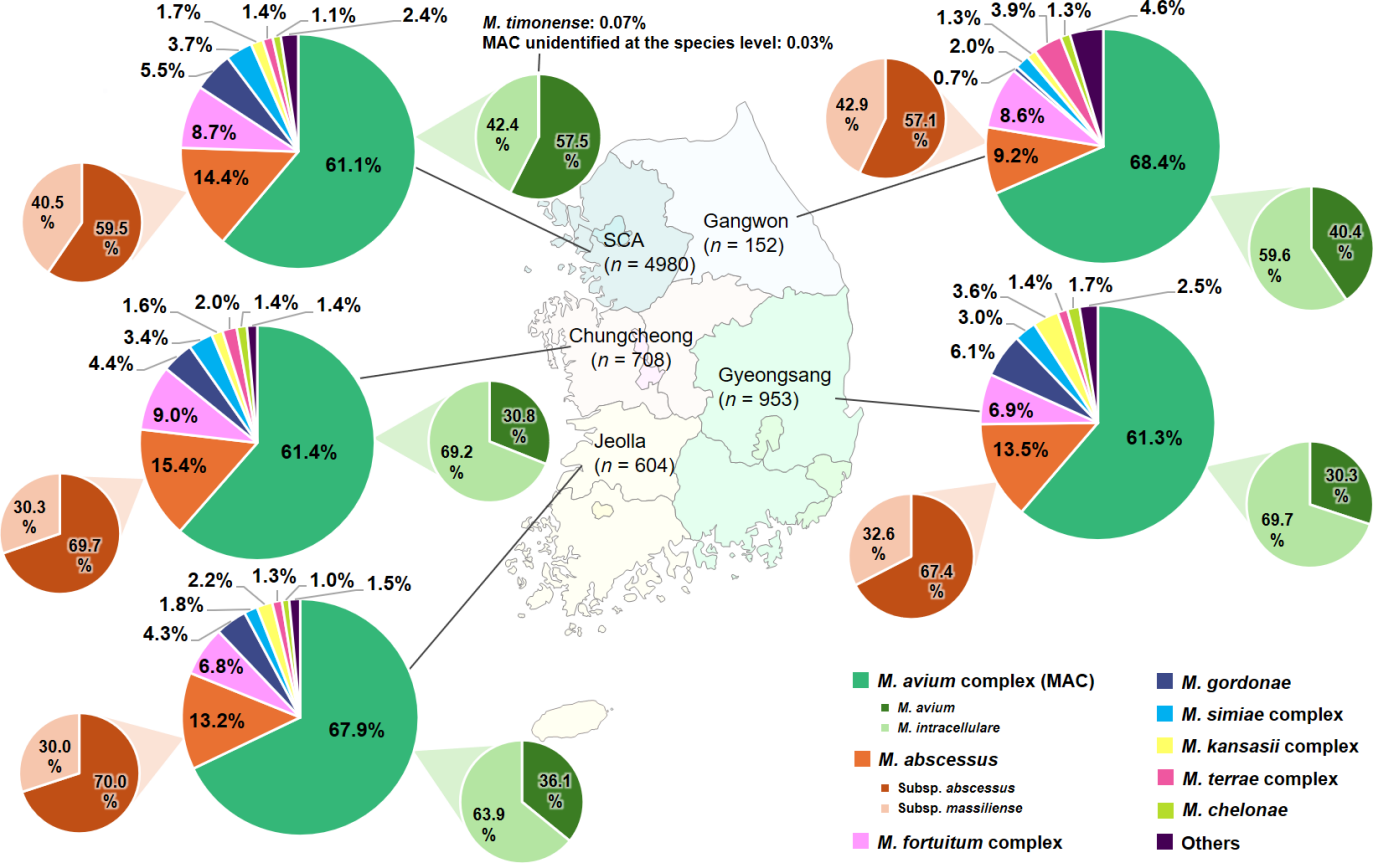
Distribution of the eight major NTM species across five regions of South Korea.

The geographical distribution of the eight major species is depicted in Fig. 4. *M. avium* complex was the most commonly isolated species across all five regions, representing 61.1% to 68.4% of all NTM isolates. It was followed by *M. abscessus*, accounting for 9.2% to 15.4%, and the *M. fortuitum* complex, with proportions of 6.8% to 9.0%. The relative ratio of *M. avium* to *M. intracellulare* was higher in the northern regions (SCA: 1.36; Gangwon: 0.68) than in the southern regions (Chungcheong: 0.45; Gyeongsang: 0.43; Jeolla: 0.56). In contrast, the relative ratio of *M. abscessus* subsp. *abscessus* to *M. abscessus* subsp. *massiliense* was higher in the southern regions (Chungcheong: 2.30; Gyeongsang: 2.07; Jeolla: 2.33) than in the northern regions (SCA: 1.47; Gangwon: 1.33).

NTM isolates other than the eight major species accounted for 2.3% (*n* = 169) of all NTM isolates, with 161 classified into 24 species and groups. Among these, 17 (70.8%) had proportions below 0.1%. Eight isolates could not be identified at the species or group level despite multigene sequencing (Fig. 2), with their closest species, as determined by sequence analysis, listed in Table 1. Two of these isolates (SMC-2 and SMC-4) underwent WGS. Genomic analysis revealed that the genome of strain SMC-2 consists of a 5,978,569 bp circular chromosome and a 49,197 bp circular plasmid (Fig. S1), with a total size of 6,027,766 bp and a DNA G+C content of 67.6%. Genome annotation predicted a total of 5,694 genes, including 5,639 protein-coding genes, 50 tRNA genes, 3 rRNA genes, and 2 tmRNA genes. The genome of strain SMC-4 comprises a 5,584,205 bp circular chromosome, two circular plasmids of 134,412 bp and 25,902 bp (Fig. S2), and eight linear contigs ranging from 1,489 bp to 70,493 bp, with a total genome size of 5,936,685 bp and a DNA G+C content of 66.6%. Genome annotation predicted 5,700 genes, including 5,644 protein-coding genes, 49 tRNA genes, 6 rRNA genes, and 1 tmRNA gene. ANI and dDDH values between these two isolates and closely related *Mycobacterium* type strains ranged from 82.5% to 89.5% and 25.4% to 38.6% for SMC-2 (Table 2) and from 76.8% to 83.9% and 20.1% to 27.1% for SMC-4 (Table 3), respectively, all below the species delineation thresholds (95%–96% for ANI and 70% for dDDH) (48).

**TABLE 1:** Sequence analysis results of eight isolates that could not be identified at the species or group level

Strain	Sequence similarity (%)		
	16S rRNA gene	*rpoB* gene	*hsp65* gene
SMC-1	1. *M. scrofulaceum* (99.4) 2. *M. mantenii* (99.4) 3. *M. paraffinicum* (99.2)	1. *M. europaeum* (95.9) 2. *M. parascrofulaceum* (95.2) 3. *M. scrofulaceum* (94.8)	1. *M. paraense* (96.8) 2. *M. parmense* (96.3) 3. *M. avium* subsp. *avium* (96.0)
SMC-2	1. *M. nebraskense* (99.8) 2. *M. paraseoulense* (99.4) 3. *M. seoulense* (99.4)	1. *M. seoulense* (96.0) 2. *M. paraseoulense* (95.7) 3. *M. nebraskense* (95.4)	1. *M. paraense* (97.5) 2. *M. parmense* (97.0) 3. *M. malmoense* (96.5)
SMC-3	1. *M. montefiorense* (99.0) 2. *M. saskatchewanense* (98.6) 3. *M. helveticum* (98.6)	No amplification	1. *M. parmense* (96.0) 2. *M. palustre* (95.8) 3. *M. avium* subsp. *avium* (95.8)
SMC-4	1. *M. goodii* (97.9) 2. *M. duvalii* (97.7) 3. *M. smegmatis* (97.5)	1. *M. neoaurum* (92.0) 2. *M. aurum* (91.5) 3. *M. monacense* (91.0)	1. *M. vanbaalenii* (96.3) 2. *M. psychrotolerans* (95.8) 3. *M. chlorophenolicum* (95.5)
SMC-5	1. *M. asiaticum* (98.8) 2. *M. marinum* (98.1) 3. *M. ulcerans* (98.1)	1. *M. gordonae* (96.1) 2. *M. intermedium* (95.4) 3. *M. asiaticum* (92.5)	No amplification
SMC-6	1. *M. mengxianglii* (98.6) 2. *M. litorale* (98.6) 3. *M. flavescens* (98.4)	1. *M. baixiangningiae* (99.4) 2. *M. litorale* (98.4) 3. *M. mageritense* (97.5)	1. *M. litorale* (97.5) 2. *M. monacense* (94.8) 3. *M. doricum* (94.3)
SMC-7	1. *M. branderi* (99.4) 2. *M. shimoidei* (96.9) 3. *M. celatum* (96.0)	1. *M. cookii* (94.3) 2. *M. conspicuum* (94.3) 3. *M. talmoniae* (93.6)	1. *M. fragae* (94.3) 2. *M. gastri* (94.0) 3. *M. persicum* (94.0)
SMC-9	1. *M. confluentis* (98.2) 2. *M. mengxianglii* (98.2) 3. *M. hubeiense* (97.8)	No amplification	1. *M. chitae* (95.8) 2. *M. brumae* (93.5) 3. *M. insubricum* (92.8)

**TABLE 2:** ANI and dDDH values between strain SMC-2 and related type strains within the genus *Mycobacterium*^*a*^

Strain	Accession no.	ANI (%)	dDDH (%)
*Mycobacterium* sp. SMC-2	GCA_025263265	N/A	N/A
*M. nebraskense* DSM 44803^T^	GCA_002102255	89.5	38.6
*M. paraffinicum* DSM 44181^T^	GCA_025822865	88.6	35.8
*M. seoulense* JCM 16018^T^	GCA_010731595	88.5	35.7
*M. europaeum* DSM 45397^T^	GCA_002102155	88.2	34.8
*M. paraseoulense* JCM 16952^T^	GCA_010731655	88.1	34.9
*M. parascrofulaceum* ATCC BAA-614^T^	GCA_000164135	88.1	34.5
*M. scrofulaceum* DSM 43992^T^	GCA_002086735	87.7	34.1
*M. malmoense* ATCC 29571^T^	GCA_019645855	83.5	26.7
*M. intracellulare* ATCC 13950^T^	GCA_000277125	83.4	26.5
*M. avium* subsp. *avium* DSM 44156^T^	GCA_009741445	83.4	26.4
*M. bohemicum* DSM 44277^T^	GCA_001053185	82.5	25.4

^*a*^ N/A, not applicable.

**TABLE 3:** ANI and dDDH values between strain SMC-4 and related type strains within the genus *Mycobacterium*^*a*^

Strain	Accession no.	ANI (%)	dDDH (%)
*Mycobacterium* sp. SMC-4	GCA_025263265	N/A	N/A
*M*. *duvalii* JCM 6396^T^	GCA_010726645	83.9	27.1
*M*. *vaccae* 95051^T^	GCA_001655245	79.6	22.3
*M*. *austroafricanum* DSM 44191^T^	GCA_000612725	79.5	22.2
*M*. *chlorophenolicum* NBRC 15527^T^	GCA_001552315	79.4	22.2
*M*. *psychrotolerans* JCM 13323^T^	GCA_010729305	79.3	22.1
*M*. *chubuense* NCTC 10819^T^	GCA_900453455	79.3	22.1
*M*. *rutilum* DSM 45405^T^	GCA_900108565	78.0	21.2
*M*. *litorale* JCM 17423^T^	GCA_010731695	77.6	20.7
*M*. *flavescens* DSM 43991^T^	GCA_025822705	77.6	20.7
*M*. *pulveris* JCM 6370^T^	GCA_010725725	77.3	20.4
*M*. *celeriflavum* JCM 18439^T^	GCA_010731795	76.8	20.1

^*a*^ N/A, not applicable.

## DISCUSSION

We provided estimates of NTM epidemiology in South Korea from 2017 to 2022 by analyzing laboratory data from a large tertiary care hospital serving a substantial number of NTM patients nationwide. By analyzing a large number of NTM isolates and utilizing multigene sequencing and WGS with high discriminatory power, we obtained valuable data on the distribution of rarely encountered NTM species, as well as novel *Mycobacterium* species. Considering the significant variations in clinical relevance and drug resistance patterns among NTM species (9, 17–24), the species distribution data presented in this study can assist in the precise diagnosis and effective management of NTM pulmonary disease in South Korea.

We observed a significant increase in the proportion of NTM among all mycobacterial isolates from respiratory specimens between 2017 and 2022, a trend consistent with previous reports from South Korea (11, 15, 29–33). However, the absolute number of NTM isolates did not increase, likely due to reduced patient access to tertiary care hospitals during the COVID-19 pandemic. The *M. avium* complex was the most commonly isolated NTM (61.9%), followed by *M. abscessus* (14.2%) and *M. fortuitum* complex (8.4%), in line with prior studies in the country (10, 15, 29, 31–33). The eight most frequently isolated NTM species (*M. avium* complex, *M. abscessus*, *M. fortuitum* complex, *M. gordonae*, *M. simiae* complex, *M. kansasii* complex, *M. terrae* complex, and *M. chelonae*) accounted for 97.7% of all NTM isolates, indicating that molecular assays targeting these major species can identify the vast majority of NTM isolates from respiratory specimens in South Korea. Beyond these major species, we identified 24 additional species and groups, most with proportions below 0.1%. Some species, including *M. duvalii*, *M. elephantis*, *M. holsaticum*, *M. iranicum*, and *M. shinjukuense*, have been implicated in human disease (49–52). However, commercial LPAs widely used in clinical microbiology laboratories cannot identify these rare yet clinically important species. Therefore, hospital and reference laboratories require diagnostic tools capable of reliably identifying them.

*M. avium* complex is the most frequently isolated NTM in most countries; however, species distribution within this complex varies across countries and regions within countries (8, 9, 13). In this study, the relative ratio of *M. avium* to *M. intracellulare* was higher in the northern regions (0.68–1.36) than in the southern regions (0.43–0.56), which is consistent with previous studies (10, 11, 30, 32). Compared to species distribution within *M. avium* complex, subspecies distribution within *M. abscessus* has been less frequently studied. Nevertheless, understanding this distribution is important, as *M. abscessus* subspecies differ in macrolide resistance and consequently exhibit varying responses to macrolide-based therapy (19, 53–55). In this study, the relative ratio of *M. abscessus* subsp. *abscessus* to *M. abscessus* subsp. *massiliense* was higher in the southern regions (2.07–2.33) than in the northern regions (1.33–1.47). However, previous studies reported the opposite trend, with a lower ratio in the southern regions (1.62) and a higher ratio in the northern regions (2.44–5.19) (10, 32, 33). These conflicting findings highlight the need for large-scale nationwide studies to clarify the geographical distribution of *M. abscessus* subspecies. Disparities in species distribution between northern and southern regions of South Korea may be attributed to differences in local environments, as NTM are typically acquired from environmental sources, such as water and soil (56). Further studies are needed to explore species-specific environmental niches that may account for geographical disparities in species distribution.

Compared to LPAs, gene sequencing offers higher discriminatory power and is considered the reference standard for NTM identification. Notably, supplementing 16S rRNA gene sequencing with additional targets, such as *rpoB* and *hsp65,* provides the highest discriminatory power, enabling subspecies-level identification in species, such as *M. abscessus* (57). However, despite using this method, eight NTM isolates (0.11%) remained unidentified at the species or group level. These isolates were considered likely to represent novel *Mycobacterium* species due to their low sequence similarity to existing species, and genomic analysis of two isolates confirmed this. The availability of gene sequencing and WGS has advanced the taxonomy of mycobacteria by facilitating the discovery of new species and the reclassification of existing ones (58–61). Given that the clinical relevance of novel species is often not well-established, ongoing surveillance of their isolation and the collection of clinical data from affected patients are essential.

One major limitation of our study is that it was conducted at a single center, which may not fully reflect the national NTM epidemiology of South Korea. In particular, the number of NTM isolates from regions outside the SCA was relatively low, ranging from 152 to 953 isolates per region. This likely reflects the population concentration in the SCA and limited our ability to accurately assess regional species distribution. In addition, the NTM isolation rate in respiratory specimens submitted for AFB culture at our center (19.9%) was significantly higher than the rate (3.7%) observed in a study conducted in a referral laboratory that received specimens from multiple hospitals nationwide during a similar period. The proportion of NTM among all mycobacterial isolates was also markedly higher at our center (89.6%) compared to the previous study (46.2%). These discrepancies suggest that the patient population at our center may differ substantially from those at other hospitals, making it inappropriate to generalize our findings to the national NTM epidemiology of South Korea.

Another limitation is that our study analyzed only the frequency and species distribution of NTM isolates from respiratory specimens, without assessing their clinical relevance. Particularly, as the clinical relevance of rarely encountered NTM species is often unclear, further studies are needed to clarify their role in disease. Moreover, our analysis included a relatively large number of isolates with low virulence, such as *M. gordonae* and the *M. terrae* complex, which are unlikely to be true pathogens. The annual increase in the proportion of NTM may also reflect the longer duration of treatment and follow-up required for NTM pulmonary disease compared to tuberculosis. Therefore, this trend should not be interpreted as an increase in the true incidence of NTM pulmonary disease. In addition, our approach of excluding duplicate isolates gave equal weight to isolates detected only once and those repeatedly detected, which may have led to an underestimation of the frequency of major pathogens in NTM pulmonary disease.

Furthermore, because multigene sequencing was performed only on NTM isolates unidentifiable to the species or complex level by the LPA, a full assessment of species and subspecies distribution was not possible. For instance, *M. intracellulare* subsp. *chimaera* would have been reported simply as *M. intracellulare* by the LPA and could not have been distinguished from other subspecies. Similarly, although we attempted to distinguish *M. abscessus* subsp. *abscessus* from *M. abscessus* subsp. *massiliense* by detecting the *erm* gene, *M. abscessus* subsp. *bolletii*, a rare subspecies, also carries this gene (62). Consequently, some isolates identified as *M. abscessus* subsp. *abscessus* in this study may have actually been *M. abscessus* subsp. *bolletii*. Further studies using multigene sequencing for all NTM isolates are needed to accurately determine species and subspecies distribution. Lastly, our results may be biased because species identification was performed only when requested by the attending physician.

In conclusion, our study revealed an increasing proportion of NTM, with *M. avium* complex and *M. abscessus* remaining predominant. We also observed regional disparities in species distribution within *M. avium* complex and subspecies distribution within *M. abscessus*. Additionally, we identified 24 rarely encountered species and groups, along with two strains that likely represent novel *Mycobacterium* species. While we did not assess the epidemiology of NTM pulmonary disease, our findings enhance the understanding of the evolving epidemiology of NTM isolates from respiratory specimens in South Korea. This may aid in optimizing diagnostic strategies and selecting appropriate treatment options, ultimately contributing to efforts to address the global rise in NTM incidence and to develop effective treatment strategies.

## Data Availability

The whole-genome sequences of strains SMC-2 and SMC-4 have been deposited in GenBank under accession numbers GCA_025263485 and GCA_025263265, respectively.

## SUPPLEMENTAL MATERIAL

Supplemental legend spectrum.00554-25-s0001.docx Legends for Figures S1 and S2.

Figure S1 spectrum.00554-25-s0002.tif Circular maps of the chromosome and plasmid of strain SMC-2.

Figure S2 spectrum.00554-25-s0003.tif Circular maps of the chromosome and two plasmids of strain SMC-4.

ASM does not own the copyrights to Supplemental Material that may be linked to, or accessed through, an article. The authors have granted ASM a non-exclusive, world-wide license to publish the Supplemental Material files. Please contact the corresponding author directly for reuse.
