# Supplementary material for: Species distribution of nontuberculous mycobacteria isolated from respiratory specimens at a tertiary care hospital in South Korea, 2017–2022
Source: Microbiol Spectr. 2025 Sep 17;13(10):e00554-25. doi: 10.1128/spectrum.00554-25 (PMC12502702; doi:10.1128/spectrum.00554-25)
Supplement: Supplemental legend — Legends for Figures S1 and S2. [file spectrum.00554-25-s0001.docx]

Fig. S1. Circular maps of the chromosome and plasmid of strain SMC-2. The outermost rings (1 and 2) show protein-coding genes (blue), tRNA genes (orange), tmRNA genes (brown), and rRNA genes (dark purple) on the forward and reverse strands. Ring 3 depicts the GC content (black), ring 4 illustrates the GC skew (green for positive GC skew, purple for negative GC skew), and ring 5 displays the genomic coordinates.

Fig. S2. Circular maps of the chromosome and two plasmids of strain SMC-4. The outermost rings (1 and 2) show protein-coding genes (blue), tRNA genes (light green), tmRNA genes (brown), and rRNA genes (orange) on the forward and reverse strands. Ring 3 depicts the GC content (black), ring 4 illustrates the GC skew (green for positive GC skew, purple for negative GC skew), and ring 5 displays the genomic coordinates.
