# Supplementary figures and images for: Species distribution of nontuberculous mycobacteria isolated from respiratory specimens at a tertiary care hospital in South Korea, 2017–2022
Source: Microbiol Spectr. 2025 Sep 17;13(10):e00554-25. doi: 10.1128/spectrum.00554-25 (PMC12502702; doi:10.1128/spectrum.00554-25)

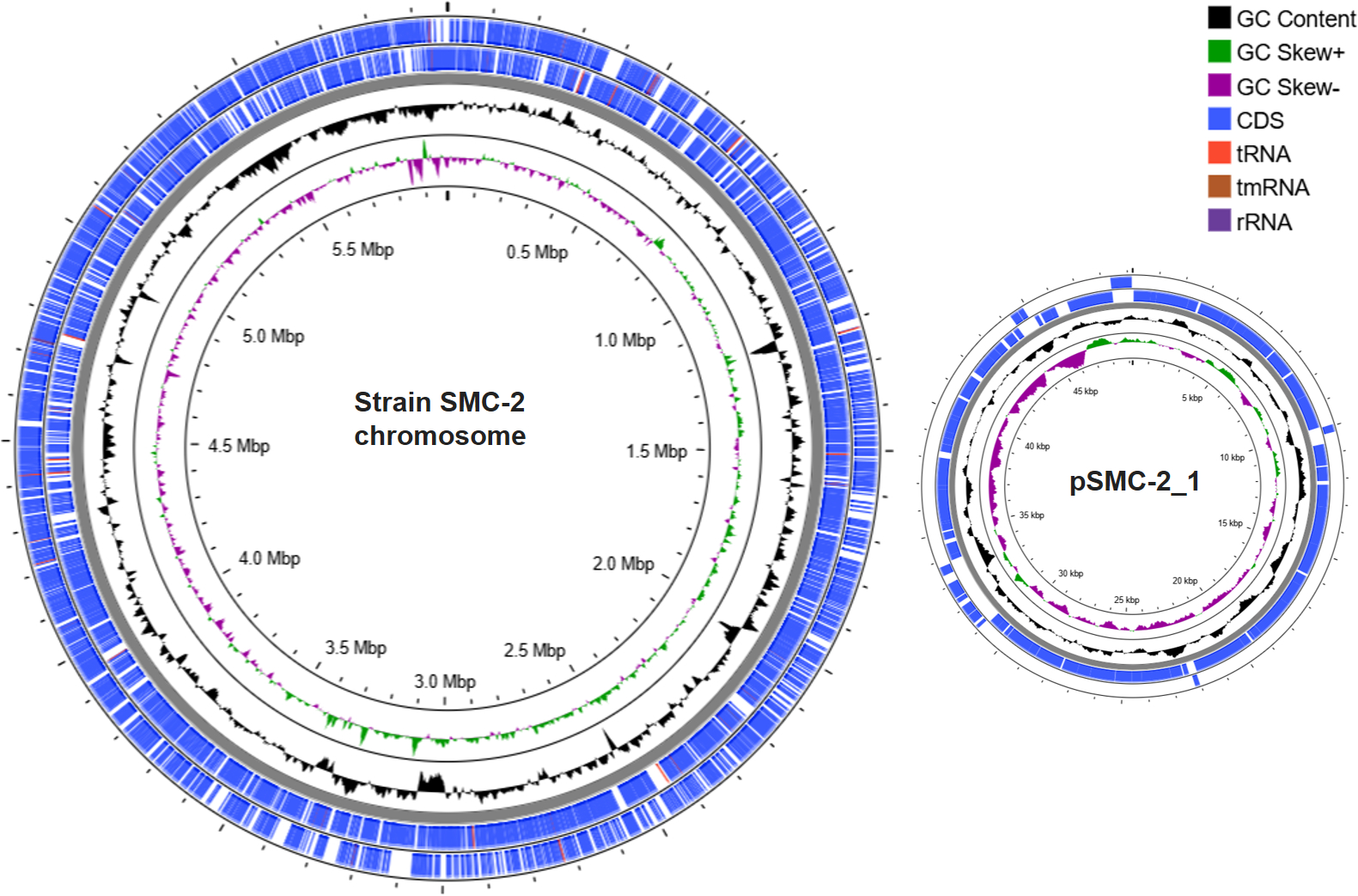

Supplement: Figure S1 — Circular maps of the chromosome and plasmid of strain SMC-2. [file spectrum.00554-25-s0002.tif]

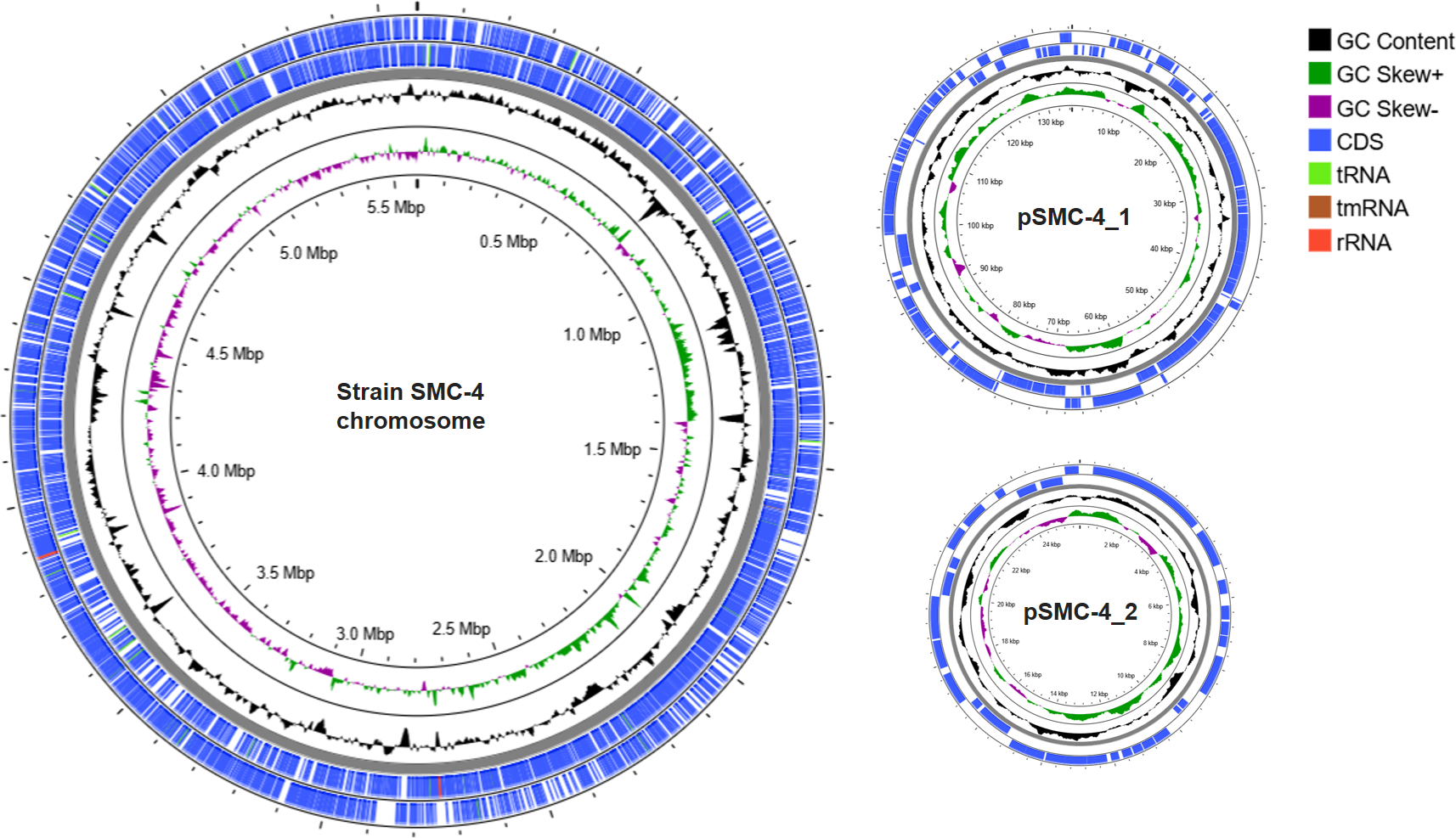

Supplement: Figure S2 — Circular maps of the chromosome and two plasmids of strain SMC-4. [file spectrum.00554-25-s0003.tif]
